# SARS‐CoV‐2 infection results in upregulation of Plasminogen Activator Inhibitor‐1 and Neuroserpin in the lungs, and an increase in fibrinolysis inhibitors associated with disease severity

**DOI:** 10.1002/jha2.654

**Published:** 2023-02-23

**Authors:** Kevin H. Toomer, Gloria F. Gerber, Yifan Zhang, Laetitia Daou, Michael Tushek, Jody E. Hooper, Ivo M. B. Francischetti

**Affiliations:** ^1^ Department of Pathology Johns Hopkins University School of Medicine Baltimore Maryland USA; ^2^ Division of Hematology Department of Medicine Johns Hopkins University School of Medicine Baltimore Maryland USA; ^3^ Department of Biostatistics Johns Hopkins University Bloomberg School of Public Health Baltimore Maryland USA; ^4^ Department of Pathology Stanford University School of Medicine Palo Alto California USA

**Keywords:** cystatin, disseminated intravascular coagulation, fibrinolysis, histidine‐rich glycoprotein, kazal, SARS‐CoV‐2, serpin, thrombophilia, thrombosis

## Abstract

Severe acute respiratory syndrome coronavirus 2 (SARS‐CoV‐2) infection results in coagulation activation although it is usually not associated with consumption coagulopathy. D‐dimers are also commonly elevated despite systemic hypofibrinolysis. To understand these unusual features of coronavirus disease 2019 (COVID‐19) coagulopathy, 64 adult patients with SARS‐CoV‐2 infection (36 moderate and 28 severe) and 16 controls were studied. We evaluated the repertoire of plasma protease inhibitors (Serpins, Kunitz, Kazal, Cystatin‐like) targeting the fibrinolytic system: Plasminogen Activator Inhibitor‐1 (PAI‐1), Tissue Plasminogen Activator/Plasminogen Activator Inhibitor‐1 complex (t‐PA/PAI‐1), α‐2‐Antiplasmin, Plasmin‐α2‐Antiplasmin Complex, Thrombin‐activatable Fibrinolysis Inhibitor (TAFI)/TAFIa, Protease Nexin‐1 (PN‐1), and Neuroserpin (the main t‐PA inhibitor of the central nervous system). Inhibitors of the common (Antithrombin, Thrombin‐antithrombin complex, Protein Z [PZ]/PZ inhibitor, Heparin Cofactor II, and α2‐Macroglobulin), Protein C ([PC], Protein C inhibitor, and Protein S), contact (Kallistatin, Protease Nexin‐2/Amyloid Beta Precursor Protein, and α‐1‐Antitrypsin), and complement (C1‐Inhibitor) pathways, in addition to Factor XIII, Histidine‐rich glycoprotein (HRG) and Vaspin were also investigated by enzyme‐linked immunosorbent assay. The association of these markers with disease severity was evaluated by logistic regression. Pulmonary expression of PAI‐1 and Neuroserpin in the lungs from eight post‐mortem cases was assessed by immunohistochemistry. Results show that six patients (10%) developed thrombotic events, and mortality was 11%. There was no significant reduction in plasma anticoagulants, in keeping with a compensated state. However, an increase in fibrinolysis inhibitors (PAI‐1, Neuroserpin, PN‐1, PAP, and t‐PA/PAI‐1) was consistently observed, while HRG was reduced. Furthermore, these markers were associated with moderate and/or severe disease. Notably, immunostains demonstrated overexpression of PAI‐1 in epithelial cells, macrophages, and endothelial cells of fatal COVID‐19, while Neuroserpin was found in intraalveolar macrophages only. These results imply that the lungs in SARS‐CoV‐2 infection provide anti‐fibrinolytic activity resulting in a shift toward a local and systemic hypofibrinolytic state predisposing to (immuno)thrombosis, often in a background of compensated disseminated intravascular coagulation.

**List of Abbreviations**
A1AT: α‐1‐Antitrypsin
A2AP: α‐2‐Antiplasmin
A2M: α2‐Macroglobulin
AT: Antithrombin
C1‐INH: C1‐Inhibitor
DIC: disseminated intravascular coagulation
ELISA: enzyme‐linked immunosorbent assay
FXIII: Factor XIII
HCII: Heparin Cofactor II
HRG: Histidine‐rich glycoprotein
ICU: intensive care unit
PAI‐1: Plasminogen Activator Inhibitor‐1
PAP: Plasmin‐α2‐Antiplasmin Complex
PC: Protein C
PCI: Protein C Inhibitor
PN‐1: Protease Nexin‐1
PN2/AβPP: Protease Nexin‐2/Amyloid Beta Precursor Protein
PS: Protein S
PZ: Protein Z
PZI: Protein Z Inhibitor
TAFI: Thrombin‐activatable Fibrinolysis Inhibitor
TAT: Thrombin‐antithrombin complex
TF: tissue factor
t‐PA/PAI‐1: Tissue Plasminogen Activator/Plasminogen Activator Inhibitor‐1 complex

## 1 INTRODUCTION

Severe acute respiratory syndrome coronavirus 2 (SARS‐CoV‐2) infection is associated with a coagulation disorder, complement activation, and endotheliopathy collectively described as immunothrombosis [1, 2, 3, 4]. The lungs play a major role in this process through the expression of tissue factor (TF), loss of anticoagulants, thrombomodulin and EPCR [5, 6, 7], neutrophil extracellular trap (NET) formation, and complement activation [1, 2, 3, 4]. These pro‐thrombotic events are exacerbated by elements of Virchow's triad, which engender a higher risk of developing thrombosis [1, 2, 3, 4]. However, somewhat surprisingly, these patients generally do not develop a consumption coagulopathy and plasma anticoagulants remain relatively stable. Accordingly, this picture is consistent with compensated disseminated intravascular coagulation (DIC), as opposed to the acute DIC typically seen in sepsis [8, 9, 10]. In order to understand these paradoxes, we studied the repertoire of protease inhibitors of the Serpin, Kunitz, Kazal, and Cystatin‐like families targeting the coagulation and complement cascades in the context of coronavirus disease 2019 (COVID‐19) infection.

It has also become evident that an imbalance between profibrinolytic and antifibrinolytic activity, with fibrinolysis shutdown, is an important mechanism underlying the hypercoagulable state of SARS‐CoV‐2 infection. This interpretation is based on several studies with thromboelastography and an association between the hypofibrinolytic state (e.g., high Plasminogen Activator Inhibitor‐1 [PAI‐1] levels) and thrombotic complications [10, 11, 12, 13, 14, 15, 16, 17]. However, paradoxically, these patients generally present with high D‐dimers. It is also poorly understood whether the lungs contribute to immunothrombosis through the expression of fibrinolysis inhibitors such as PAI‐1 and Neuroserpin. Moreover, it remains unclear to what extent fibrinolysis inhibitors other than PAI‐1, including Neuroserpin and Protease Nexin‐1 (PN‐1), as well as fibrinolysis modulators such as Histidine‐rich glycoprotein (HRG), contribute to coagulopathy in the infection. Therefore, we studied plasma inhibitors of fibrinolysis and the pulmonary expression of PAI‐1 and Neuroserpin. Our results are in keeping with the primary role of hypofibrinolysis in hemostasis dysregulation through different mechanisms in SARS‐CoV‐2 infection, often associated with compensated DIC.

## 2 MATERIALS AND METHODS

### 2.1 Study design and participants

Cross‐sectional study of 64 adult (≥18 years) patients with moderate disease (referred to as “moderate disease group”, *n* = 36) and severe or critical disease (referred to as “severe disease group”, *n* = 28), based on World Health Organization guidelines [18], with clinical and/or radiologic indications for hospitalization at the Johns Hopkins Hospital (period April 2020–October 2020), as reported [5]. All patients had a confirmed diagnosis of COVID‐19 by polymerase chain reaction (PCR) assays on nasopharyngeal swab samples, with the alpha variant as the most prevalent strain in this Hospital for the studied period. An additional 16 individuals negative for COVID‐19 were included as controls. Controls were patients in the Hospital for routine evaluation with no major health concerns, except for a few with obesity. Patient characteristics were recorded in Electronic Medical Records, with three authors (Kevin H. Toomer, Laetitia Daou, and Ivo M. B. Francischetti) having access to data. All experiments using human material were performed in accordance with Institutional guidelines (IRB00257218) and the agreement of the Ethical Committee of the Johns Hospital University School of Medicine. The Institutional Review Board approved this study and waived the need for consent.

### 2.2 Procedures

Blood samples were collected into Vacutainer tubes (BD, Franklin Lakes, New Jersey) containing sodium citrate (3.2%) as described [5].

### 2.3 Reagents

All hematologic parameters were performed at our Institution's clinical laboratory Siemens CS‐5100 (Siemens; Malvern, PA, USA) with manufacturer's reagents and controls per laboratory protocols. DUOSet enzyme‐linked immunosorbent assay (ELISA) performed for Kallistatin (SERPINA4, DY1669), Plasminogen Activator Inhibitor‐1 (PAI‐1, SERPINE1; DY1786), Protein Z Inhibitor (PZI, SERPINA10; DY8115), α2‐Macroglobulin (A2M; DY1938), Protease Nexin‐2/Amyloid Beta Precursor Protein (PN2/AβPP; DY850) and C1‐Inhibitor (C1‐INH, SERPING1; DY2488) from Bio‐Techne/R&D (Minneapolis, MN). ELISA for Antithrombin (AT, SERPINC1, EA3301‐1), Thrombin‐antithrombin complex (TAT; ET1020‐1), α‐2‐Antiplasmin (A2AP, SERPINF2, EA3477‐1), Plasmin‐α2‐Antiplasmin Complex (PAP; EP1807‐1), Tissue Plasminogen Activator/Plasminogen Activator Inhibitor‐1 complex (t‐PA/PAI‐1; EP1105‐1), Factor XIII (FXIII; EF1013‐1), α‐1‐Antitrypsin (A1AT, SERPINA1; EA5101‐1), Protein Z (PZ; EP3333‐1), Protein C (PC, EP2312‐1), Protein S (total) (PS, EP133‐1), and Vaspin (SERPIN12; EV3005‐1) from Assaypro, LLC (St. Charles, MO). ELISA for HRG (EKE60289) was from Biomatik (Wilmington, DE). ELISA for Heparin Cofactor II (HCII, SERPIND1; EH414RB) and PC Inhibitor (PCI, SERPINA5; EH412RB) were from ThermoFisher/Invitrogen (Waltham, MA). ELISA for Thrombin‐activatable Fibrinolysis inhibitor (TAFI; HTAFIK) was from Molecular Innovations (Novi, MI), and activated TAFI (TAFIa; MBS3801463) was from MyBioSource, Inc. (San Diego, CA). ELISA for PN‐1 (SERPINE2; LS‐F9936) was from LSBIO (Seattle, WA). ELISA for Neuroserpin (SERPINI1; ab283539) was from Abcam (Boston, MA).

### 2.4 ELISA

ELISA was performed according to the manufacturers’ instructions and Synergy HTX Multi‐mode Microplate Reader, interfaced with Gen5 2.09 Software (BioTek Instruments, VT), as described [5].

### 2.5 Autopsy

All autopsies were consented for by legal next of kin and performed by the Autopsy Service at the Johns Hopkins Hospital as described [19].

### 2.6 Immunohistochemistry studies

Immunohistochemical (IHC) staining was carried out at the Oncology Tissue Services Core of the Johns Hopkins University School of Medicine. Immunolabeling for all antigens was performed on formalin‐fixed, paraffin‐embedded sections on a Ventana Discovery Ultra autostainer (Roche Diagnostics) [5]. Following dewaxing and rehydration on board, epitope retrieval was performed using Ventana Ultra CC1 buffer (Tris‐EDTA buffer pH 7.8, Roche Diagnostics) at 96°C for 64 min. Primary antibodies, including rabbit polyclonal anti‐PAI‐1 (1:200, ab66705; Abcam), rabbit polyclonal anti‐Neuroserpin (1:250, PA‐5110636; ThermoFisher), and rabbit polyclonal anti‐TF (1:400; PA‐27278; ThermoFisher) were applied at 36°C for 60 min, and detected using an anti‐rabbit HQ detection system. This step was followed by a Chromomap DAB IHC detection kit (Roche Diagnostics), counterstaining with Mayer's hematoxylin, dehydration, and mounting. Other primary antibodies were: anti‐CD68 for macrophages, AE1/AE3 (cytokeratin cocktail) for epithelial cells, and anti‐CD34 for endothelial cells (detected by routine automated staining). For each case, one IHC slide was independently semi‐quantitatively scored by two pathologists (Kevin H. Toomer and Ivo M. B. Francischetti) with an excellent agreement, as follows: rare or absent (0), ≤ 10% (1+), 11–25% (2+), 26–50% (3+), > 51% (4+). Mann‐Whitney *U* test was employed for statistical analysis.

### 2.7 Statistical analysis

Descriptive statistics as described [5].

## 3 RESULTS

### 3.1 Demographics

Sixty‐four adult patients (36 with moderate and 28 with severe disease) with PCR‐confirmed SARS‐CoV‐2 were included in our cohort. Sixteen individuals were selected as controls (Table 1). Age was the only variable to show a statistically significant difference between moderate and severe cases (49 years old vs. 68 years old, *p* < 0.001), while sex, body mass index, and comorbidities did not differ. The most common comorbidities for both groups combined were obesity (50%), hypertension (34%), diabetes (31%), and asthma (23%). Other less common comorbidities included a history of venous thrombosis and chronic kidney disease. All COVID‐19 patients were on prophylactic dose anticoagulation, according to guideline recommendations for standard intensity or high‐intensity anticoagulation based on patient risk profile. Almost all patients (98%) were on low molecular weight heparin (enoxaparin) or unfractionated heparin, with the remainder on warfarin or direct oral anticoagulants. Two patients (5.6%) in the moderate disease group developed pulmonary embolisms. Four patients in the severe disease group developed venous, arterial thrombosis or PE, and 16 (57%) were admitted to the Intensive Care Unit (ICU). Overall, the incidence of thrombosis in all COVID‐19 patients was about 10%. There were seven deaths in the severe disease group (25%), corresponding to 11% overall mortality.

**TABLE 1:** Demographics, laboratory, anticoagulant use, clinical and outcome information for cohort cases

	**Control (*n* = 16)**	**Moderate (*n* = 36)**	**Severe (*n* = 28)**	***p*‐Value** *
**Demographics**				
Sex, male	7 (43.8)	14 (38.9)	10 (35.7)	NS^a^
Age, years	60.5 [52.8–66.3]	49 [37–66.3]	68 [60–78]	0.006^b^
BMI, Kg/m^2^	30 [24.3–33.6]	29.1 [25.8‐37.4]	30.7 [26.0–35.1]	NS^b^
Comorbidities	10 (62.5)	32 (88.8)	26 (92.9)	NS^a^
**Comorbidities**				
Obesity	8 (50.0)	17 (47.2)	15 (53.6)	NS^a^
Hypertension	4 (25.0)	10 (27.8)	12 (42.9)	NS^a^
Diabetes	0 (0)	8 (22.2)	12 (42.9)	NS^a^
Asthma	1 (6.3)	10 (27.8)	5 (17.9)	NS^a^
Coronary artery disease, MI, TIA, stroke	3 (18.8)	4 (11.1)	8 (28.6)	NS^a^
Venous thrombosis or pulmonary embolism	0 (0)	2 (5.6)	2 (7.1)	NS^a^
Chronic kidney disease	0 (0)	5 (13.9)	7 (25)	NS^a^
**Routine laboratory tests**				
Hemoglobin (g/dl)	13.0 [12.5–13.3]	11.7 [10.8–13.3]	11.3 [9.8–12.8]	0.024^b^
White blood cell (K/μl)	6.3 [5.6–6.9]	5.8 [4.4–8.0]	8.4 [5.1–10.9]	0.037^b^
Absolute monocyte count (K/μl)	0.58 [0.55–0.65]	0.38 [0.31–0.56]	0.45 [0.33–0.63]	NS^b^
Absolute lymphocyte count (K/μl)	1.3 [1.1–2.0]	1.1 [0.9–1.6]	1.0 [0.7–1.4]	NS^b^
Absolute neutrophil count (K/μl)	3.2 [2.7–3.8]	4.1 [3.2–5.3]	6.7 [3.8–8.9]	0.005^b^
C‐reactive protein (mg/dl)	Ref. (0–4.9)	5.1 [2.1–9.7]	10.4 [2.5–15.0]	NS^c^
Plasma creatinine (mg/dl)	Ref. (0.6–1.3)	0.8 [0.6–1.2]	1.0 [0.8–1.3]	0.016^c^
Lactate dehydrogenase (U/L)	Ref. (84–197)	317.0 [250.5–431.5]	369.0 [315.0–836.0]	NS^c^
Ferritin (ng/ml)	Ref. (13–150)	398.0 [125.0–845.0]	847.0 [481.5–1920.5]	0.012^c^
Interleukin‐6 (pg/ml)	Ref. (<10)	31.5 [18.4–71.9]	41.6 [24.2–109.8]	NS^c^
**Coagulation**				
PT (sec)	10.6 [10.4–10.9]	10.9 [10.6–11.4]	11.3 [10.9–11.8]	0.0004^b^
aPTT (sec)	27.0 [24.6–30.0]	31.5 [28.6–32.9]	32.0 [28.3–34.5]	0.012^b^
Fibrinogen (mg/dl)	Ref. (170–422)	475.5 [404.0–574.0]	551.5 [381.5–683.8]	NS^c^
Platelets (K/μl)	226 [188–286]	195 [162–268]	236 [183–284]	NS^b^
D‐dimers (μg/ml)	Ref. (0.00–0.49)	0.73 [0.44–1.21]	1.14 [0.81–2.16]	0.025^c^
DIC score	0 (0)	1.0 (0.29)	1.6 (0.31)	NS^c^
**Anticoagulants**				
LMWH (enoxaparin)	–	28 (77.8)	22 (78.6)	NS^a^
Standard Heparin	–	6 (16.7)	7 (25)	NS^a^
Warfarin	–	1 (2.8)	1 (3.6)	NS^a^
Rivaroxaban or Apixaban	–	1 (2.8)	4 (14.3)	NS^a^
**Thrombosis (during hospitalization)**				
Venous Thrombosis	0 (0)	0 (0)	1 (3.6)	NS^a^
Pulmonary Embolism	0 (0)	2 (5.6)	2 (7.1)	NS^a^
Coronary artery disease, MI, TIA, stroke	0 (0)	0 (0)	1 (3.6)	NS^a^
**Hospital stay and Outcome**				
ICU stay	0	0	16 (57.1)	0.0001^a^
Death	0	0	7 (25)	0.0019^a^

The value is reported as *n* (%), mean (SEM), or median [IQR].

^a^ Fisher's exact test, two‐tailed (categorical variable).

^b^ Kruskal‐Wallis test.

^c^ Mann‐Whitney *U* test (continuous variables).

* *p* values refer to the comparison between moderate and severe disease groups (Fisher's exact test and Mann‐Whitney *U* test), or between control, moderate, and severe disease groups (Kruskal‐Wallis test).

Abbreviations: BMI, body mass index; LMWH, low‐molecular‐weight heparin; MI, myocardial infarction; TIA, transient ischemic attack; ICU intensive care unit; NS, non‐significant.

### 3.2 Routine biochemical, hematologic, and coagulation parameters

Several parameters were evaluated, with the mean ± SEM or median, indicated in Table 1. Numerous hematologic parameters differentiated both groups statistically, including hemoglobin (*p* = 0.024), white blood cell count (*p* = 0.037), and absolute neutrophil count (*p* = 0.005), but not absolute monocyte count (AMC) or absolute lymphocyte count. C‐reactive protein, lactate dehydrogenase (*p* = NS), and interleukin‐6 (*p* = NS) were not statistically significant between groups, in contrast to ferritin (*p* = 0.012) and creatinine (*p* = 0.016). Table 1 shows that fibrinogen and platelets did not differ between moderate and severe disease groups, while PT (*p* = 0.0004), aPTT (*p* = 0.012), and D‐dimer (*p* = 0.025) were discriminatory. The DIC score, based on the International Society on Thrombosis and Haemostasis criteria, was similar in severe versus moderate disease groups (*p* = NS) and averaged less than two in both groups.

### 3.3 Protease inhibitors of the contact pathway

**FIGURE 1:**
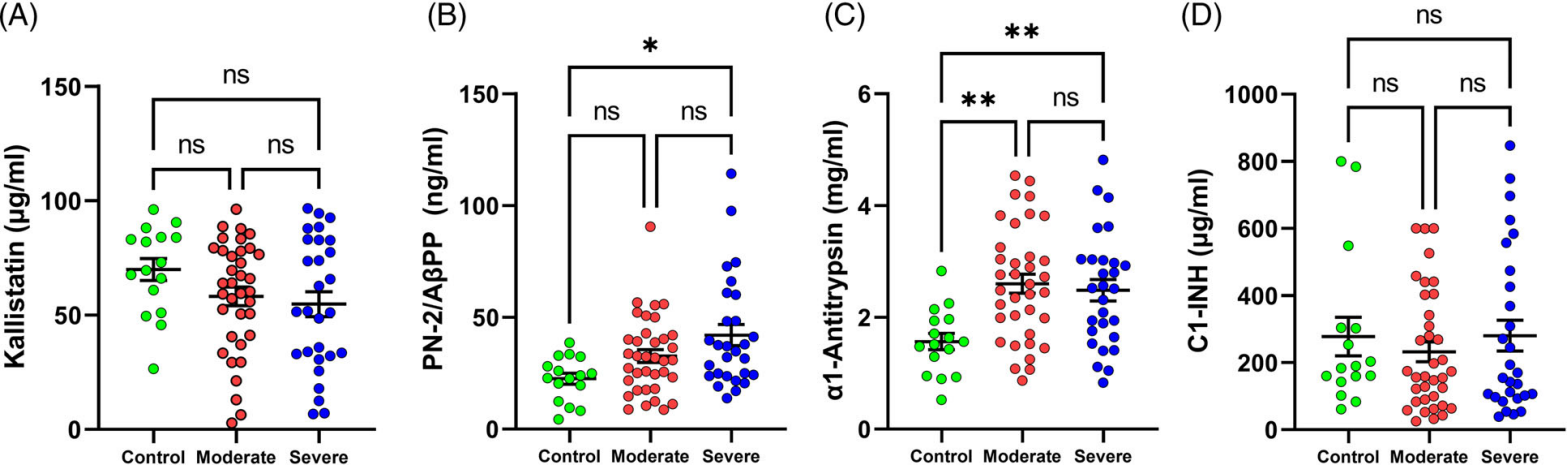
Inhibitors of the contact pathway and complement cascade in SARS‐CoV‐2 infection and controls. (A) Kallistatin, (B) PN‐2/AβPP, (C) α1‐Antitrypsin, and (D) C1‐INH plasma concentrations were determined by ELISA in controls and COVID‐19 patients. Each symbol corresponds to one patient. The means and SEM are indicated. NS, non‐significant; *p ≤ 0.05; **p ≤ 0.01 (Kruskal‐Wallis test or Mann‐Whitney *U* test).

COVID‐19 infection has been associated with the formation of NETs and consumption of kallikrein and FXIIa [20, 21]. Kallikrein and FXIIa reciprocally autoactivate, leading to the activation of FXIa in the contact pathway. Kallistatin is the main inhibitor of prekallikrein, with the formation of a high affinity complex typical of serpins. No statistically significant difference (*p* = NS) was detected in Kallistatin concentration between control (69 ± 4.7 μg/ml), moderate, and severe disease (Figure 1A). FXIa plays a major role in the downstream activation of FIX and serves as an amplification step by thrombin/polyphosphate. FXIa is under the control of PN2/AβPP, a potent and specific inhibitor [22]. No difference in PN2/AβPP levels was observed between control (22.50 ± 0.4 ng/ml) and moderate disease groups, although PN2/AβPP was significantly elevated in severe cases versus controls (*p* = 0.01) (Figure 1B). SARS‐CoV‐2 infection is associated with the NET formation and high levels of neutrophil elastase [5]. α1‐antitrypsin (A1AT) is an abundant inhibitor of elastase as well as other enzymes [23]. A1AT showed an increase in moderate (*p* = 0.001) and severe (*p* = 0.009) disease versus controls (1.56 ± 0.1 mg/ml) (Figure 1C). Complement activation is a well‐described event in SARS‐CoV‐2 infection, and C1‐INH is a main inhibitor of the classical and lectin complement pathways [24]. Figure 1D shows no statistical (*p* = NS) difference in C1‐INH concentrations in moderate or severe disease versus normal controls (280 ± 50 μg/ml).

### 3.4 Protease inhibitors of the common and Protein C pathway

**FIGURE 2:**
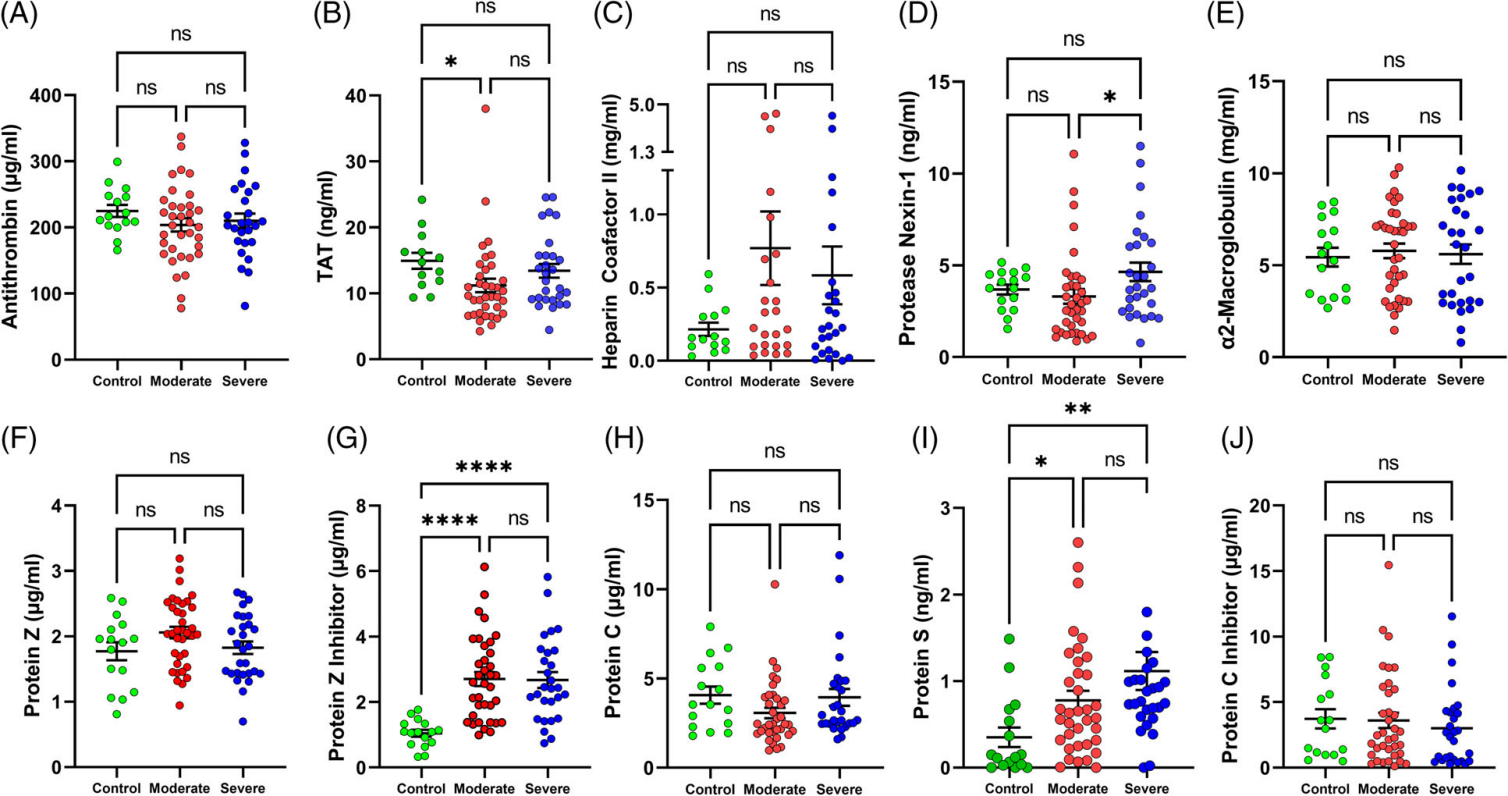
Inhibitors and cofactors of the common and Protein C pathways in SARS‐CoV‐2 infection and controls. (A) Antithrombin, (B) TAT, Thrombin‐Antithrombin complex, (C) Heparin Cofactor II, (D) Protease Nexin‐1, (E) α2‐macroglobulin, (F) Protein Z, (G) Protein Z Inhibitor, (H) Protein C, (I) Protein S (total), and (J) Protein C Inhibitor plasma concentrations were determined by ELISA in controls and COVID‐19 patients. Each symbol corresponds to one patient. Means and SEM are indicated. NS, non‐significant; *p ≤ 0.05; ****p ≤ 0.0001 (Kruskal‐Wallis test, or Mann‐Whitney *U* test).

The contact and extrinsic pathways result in the generation of thrombin, platelet aggregation, clot formation, and inflammation. There are several plasma inhibitors of thrombin. Among the most abundant is AT, which requires heparin for full inhibitory activity [23]. Our results show high concentrations of AT in controls (225 ± 35 μg/ml) with no significant change when compared to moderate and severe disease groups (*p* = NS) (Figure 2A). AT forms a high‐affinity complex with thrombin known as thrombin‐antithrombin complex (TAT). No significant increase in TAT was observed among all groups studied, with a mean TAT concentration of 14.9 ng/ml in controls (Figure 2B). HCII is a thrombin inhibitor whose activity is increased 1000‐fold by dermatan sulfate [23]. HCII concentration was higher in moderate and severe cases compared to controls (215 ± 45 μg/ml), but it did not reach statistical significance (*p* ≤ 0.05)(Figure 2C). PN‐1 is an efficient inhibitor of thrombin released by activated platelets and a negative modulator of fibrinolysis [25]. The concentration of PN‐1 in controls (3.6 ± 0.2 ng/ml) was similar in moderate and severe disease (*p* = NS), although a significant increase in severe versus moderate disease was observed (*p* = 0.038)(Figure 2D). A2M can trap many proteinases involved in coagulation and fibrinolysis including thrombin, FXa, plasmin, and kallikrein, among other enzymes, cytokines, and growth factors. A high concentration of A2M in control plasma (5.43 ± 0.5 mg/ml) remains stable in moderate and severe disease (Figure 2E). FXa is a pro‐inflammatory and pro‐coagulant enzyme of the common pathway. FXa inhibition requires the presence of PZ as a cofactor for ZPI. There were no statistically significant differences in PZ between controls (1.78 ± 0.13 μg/ml) and the other two groups (*p* = NS)(Figure 2F). However, a significant increase in PZI was found when moderate and severe disease groups (*p* ≤ 0.0001, for both) were compared to control individuals (1.04 ± 0.1 μg/ml) (Figure 2G). In the PC pathway, activated PC (aPC) functions as an anticoagulant by cleaving Factor Va and Factor VIIIa, in the presence of cofactor Protein S, being under the control of PCI [23]. Figure 2H shows that the plasma level of PC in normal individuals (4.06 ± 0.4 μg/ml) is similar to the other two groups, while there is an increase in PS (total) in these groups compared to controls (0.35 ± 0.1 ng/ml) (Figure 2I). PCI levels in moderate or severe SARS‐CoV‐2 infection were similar to normal individuals (3.73 ± 0.7 μg/ml) (Figure 2J).

### 3.5 Protease inhibitors of the fibrinolytic pathway

**FIGURE 3:**
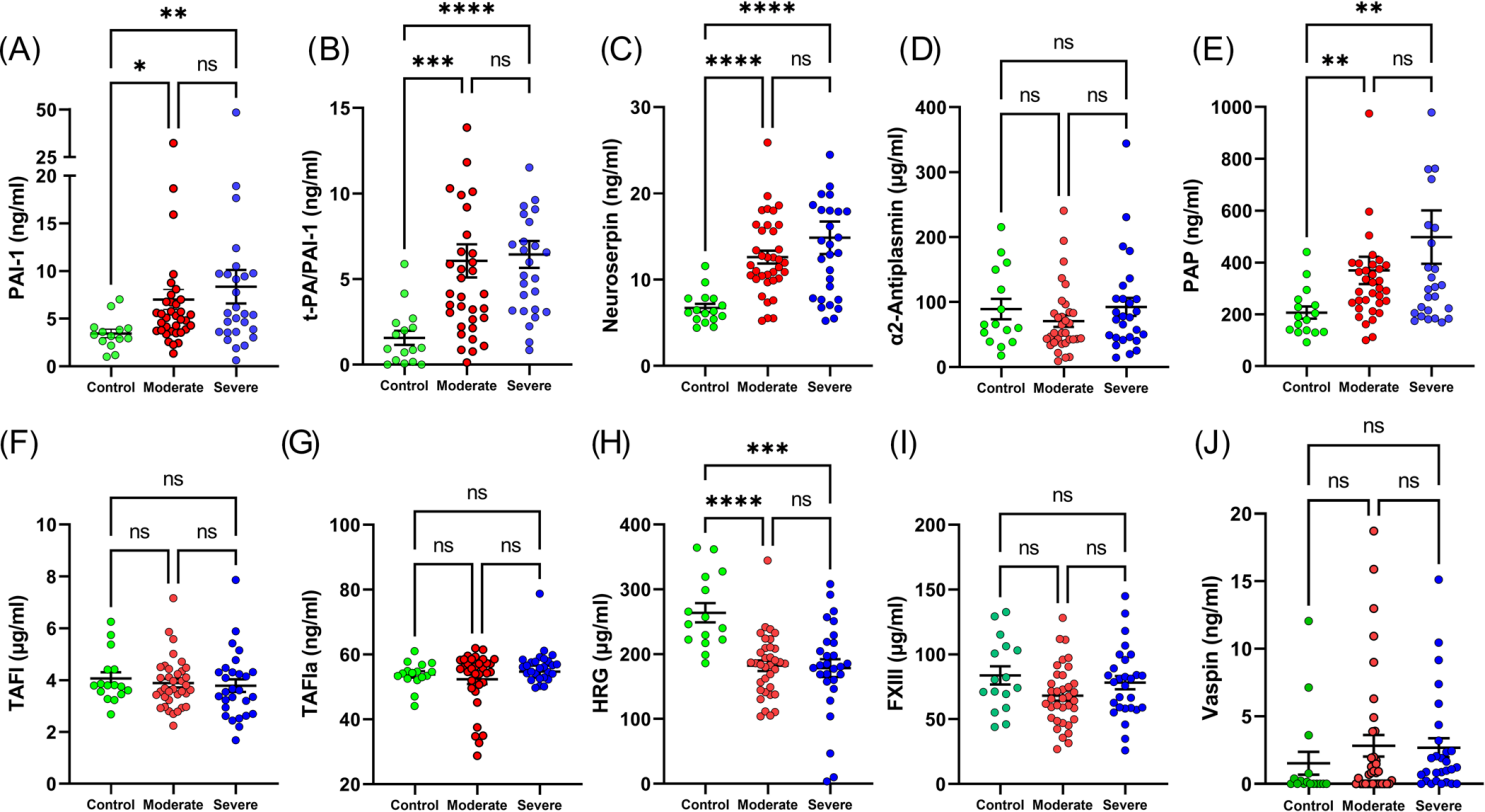
Inhibitors, components of fibrinolytic cascade and Vaspin in SARS‐CoV‐2 infection and controls. (A) PAI‐1, (B) t‐PA/PAI‐1, (C) Neuroserpin, (D) α2‐Antiplasmin, (E) PAP (Plasmin‐α2‐Antiplasmin complex), (F) TAFI, (G) TAFIa, (H) HRG (Histidine‐rich glycoprotein), (I) Factor XIII, and (J) Vaspin plasma concentrations were determined by ELISA in controls and COVID‐19 patients. Each symbol corresponds to one patient. Means and SEM are indicated. NS, non‐significant; **p ≤ 0.01, ***p ≤ 0.001 ****p ≤ 0.0001 (Kruskal‐Wallis test).

Fibrinolysis is stimulated by t‐PA, an enzyme that activates the zymogen plasminogen into the enzyme plasmin, which degrades fibrin clots [26]. PAI‐1 is the main inhibitor of t‐PA in human plasma. PAI‐1 levels were higher in patients with moderate and severe disease (*p* = 0.01 and *p* = 0.009, respectively) (Figure 3A) compared to controls (3.5 ± 0.4 ng/ml). PAI‐1 forms a complex with t‐PA in plasma, with plasma concentration of 1.6 ± 0.4 ng/ml t‐PA/PAI‐1 in controls and a significant increase for both moderate and severe disease groups (*p* ≤ 0.0003, for both) (Figure 3B). Neuroserpin is the main fibrinolytic inhibitor in the central nervous system (CNS). Neuroserpin plasma concentration of 6.72 ± 0.4 ng/ml in controls almost doubled for both groups of SARS‐CoV‐2 patients (p ≤ 0.0001) (Figure 3C). α‐2‐Antiplasmin (A2AP) is the main inhibitor of plasmin in plasma [27]. A2AP in controls (88.35 ± 14 μg/ml) remained at the same level in moderate and severe disease groups (Figure 3D). Results also show an increase in PAP complex in both moderate and severe disease (*p* ≤ 0.005) compared to COVID‐19 negative individuals (206.8 ± 23.3 ng/ml) (Figure 3E). TAFI/TAFIa is an enzymatic inhibitor of fibrinolysis, which cleaves the C‐terminal lysine necessary for tPA‐mediated plasminogen activation [26]. Figure 3F,G shows that both TAFI and its activated form, TAFIa, remained similar to controls (4.06 ± 0.2 μg/ml for TAFI, and 53.84 ± 0.9 ng/ml for TAFIa). HRG has multiple functions, including inhibition and promotion of fibrinolysis [28]. HRG levels were significantly lower in both moderate and severe disease compared to controls (263.85 ± 14.8 μg/ml) (Figure 3H). No significant changes were observed in FXIII levels in normal individuals (83.7 ± 6.9 μg/ml) compared to COVID‐19 patients (Figure 3I). Vaspin, a serpin released by adipose tissue, showed no change in COVID‐19 patients compared to controls (1.52 ± 0.8 ng/ml) (Figure 3J).

### 3.6 Association between D‐dimer levels and biomarkers of cellular activation

**FIGURE 4:**
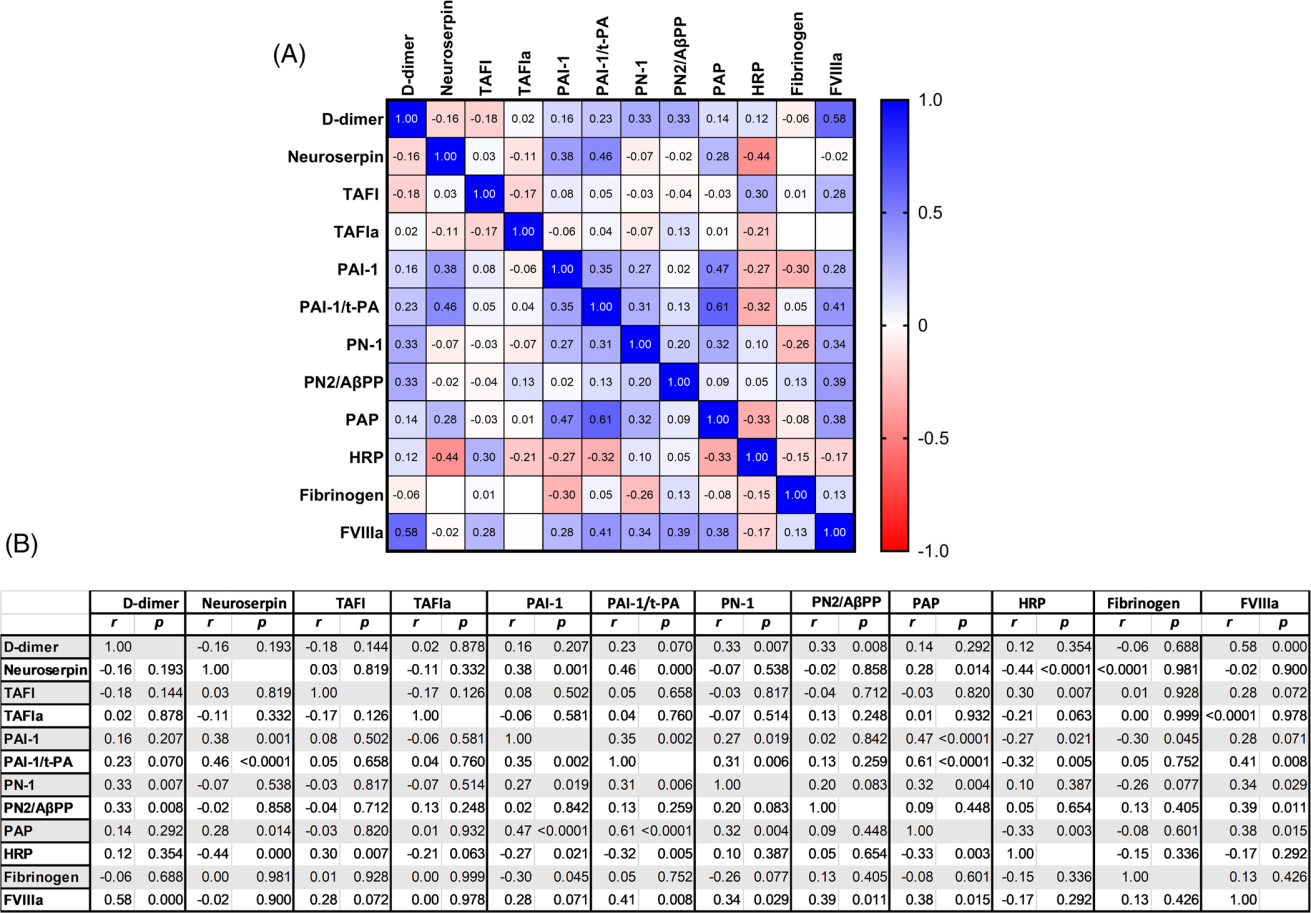
Correlation between D‐dimers, markers of fibrinolysis, FVIIIa, and fibrinogen in SARS‐CoV‐2 infection. (A) The heatmap shows positive correlations indicated by the intensity in blue, and negative correlation in red, as defined in the legend. (B) *r*‐Value and *p*‐value for each correlation are indicated. Significance set at *p* ≤ 0.05 (Spearman rank correlation coefficient).

High D‐dimers are associated with high mortality in SARS‐CoV‐2 infection [1, 2, 3, 4]. In our cohort, heat mapping showed that D‐dimers positively correlated (*p* ≤ 0.05) with PN‐1, PN2/AβPP, and FVIII. Neuroserpin positively correlated with PAI‐1, PAI‐1/t‐PA, and PAP. Additionally, FVIIIa positively correlated with t‐PA/PAI‐1, PN‐1, PN2/AβPP, and PAP. In contrast, HRG negatively correlated with Neuroserpin, TAFIa, PAI‐1, PAI‐1/t‐PA and PAP (Figure 4A). Correlations among all variables and specific *r* and *p* values are reported in Figure 4B.

### 3.7 Biomarkers associated with severe disease

A logistic regression model was generated with the outcome (control vs. moderate, control vs. severe, and moderate vs. severe) as the dependent variable, and numerous inhibitors and cofactors as independent variables. The results of the univariate analysis are shown in Table 2. Age, PN2/AβPP, A1AT, TAT, PZI, PS (total), Neuroserpin, PAI‐1, t‐PAI/PAI‐1, and HRG were significantly associated with moderate and/or severe disease on univariate regression. When a multivariate logistic regression model was employed, Age (Odds ratio, 1.04; 95% confidence interval, 1.01–1.08; *p* = 0.014) was an independent predictor of disease severity. Other variables were not independently predictive of disease severity.

**TABLE 2:** Univariate logistic regression of association of covariates with moderate and severe diseases

	**Control versus Moderate**			**Control versus Severe**			**Moderate versus Severe**		
**Variables**	**OR**	**95% CI**	***p*‐Value**	**OR**	**95% CI**	***p*‐Value**	**OR**	**95% CI**	***p*‐Value**
**Demographics**									
Age	0.97	0.93–1.01	0.116	1.03	0.99–1.07	0.195	1.05	1.02–1.08	0.004*
Sex	0.82	0.25–2.70	0.742	0.71	0.20–2.50	0.599	0.87	0.31–2.43	0.795
BMI	1.03	0.95–1.12	0.441	1.03	0.94–1.14	0.517	0.99	0.93–1.06	0.808
**Contact and complement pathways**									
Kallistatin	1	1.00–1.00	0.104	1	1.00–1.00	0.075	1	1.00–1.00	0.6
PN2/AβPP	1.06	1.00–1.11	0.039*	1.09	1.02–1.17	0.014*	1.02	1.00–1.05	0.089
α1‐Antitrypsin	1	1.00–1.00	0.003*	1	1.00–1.00	0.007*	1	1.00–1.00	0.641
C1‐INH	1	1.00–1.00	0.438	1	1.00–1.00	0.971	1	1.00–1.00	0.361
**Common pathway**									
Antithrombin	1	1.00–1.00	0.206	1	1.00–1.00	0.242	1	1.00–1.00	0.922
TAT complex	0.89	0.79–0.99	0.036*	0.92	0.82–1.04	0.176	1.07	0.98–1.17	0.152
Heparin Cofactor II	1	1.00–1.00	0.272	1	1.00–1.00	0.251	1	1.00–1.00	0.812
Protein Z Inhibitor	1	1.00–1.01	0.004*	1	1.00–1.00	0.002*	1	1.00–1.00	0.924
Protein Z	1	1.00–1.00	0.084	1	1.00–1.00	0.735	1	1.00–1.00	0.082
Protease Nexin‐1	0.92	0.69–1.21	0.546	1.26	0.90–1.77	0.185	1.24	1.00–1.53	0.048*
α2‐Macroglobulin	1	1.00–1.00	0.613	1	1.00–1.00	0.835	1	1.00–1.00	0.779
**Protein C pathway**									
Protein C inhibitor	1	1.00–1.00	0.897	1	1.00–1.00	0.423	1	1.00–1.00	0.461
Protein S	5.07	1.14–22.54	0.033*	15.3	2.34–100.02	0.004*	1.56	0.83–2.95	0.171
Protein C	1	1.00–1.00	0.09	1	1.00–1.00	0.863	1	1.00–1.00	0.124
**Fibrinolysis**									
Neuroserpin	1.87	1.32–2.65	<0.001*	1.6	1.15–2.22	0.005*	1.05	0.97–1.13	0.26
TAFI	1	1.00–1.00	0.538	1	1.00–1.00	0.452	1	1.00–1.00	0.723
TAFIa	0.97	0.89–1.06	0.504	1.01	0.94–1.09	0.732	1.03	0.97–1.10	0.316
PAI‐1	1.71	1.09–2.68	0.019*	1.49	1.05–2.10	0.025*	1.02	0.96–1.09	0.496
t‐PA/PAI‐1 complex	1.87	1.22–2.89	0.004*	2.29	1.36–3.85	0.002*	1.02	0.92–1.12	0.769
α2‐antiplasmin	1	1.00–1.00	0.291	1	1.00–1.00	0.838	1	1.00–1.00	0.191
PAP complex	1.01	1.00–1.02	0.008*	1.01	1.00–1.02	0.028*	1	1.00–1.00	0.266
HRG	1	1.00–1.00	0.002*	1	1.00–1.00	0.003*	1	1.00–1.00	0.796
Factor XIII	1	1.00–1.00	0.048*	1	1.00–1.00	0.507	1	1.00–1.00	0.121
**Others**									
Vaspin	1.11	0.94–1.30	0.218	1.13	0.92–1.38	0.236	0.98	0.92–1.05	0.598

CI, confidence interval; OR, odds ratio. The OR indicates the estimated change in the odds of disease severity per unit increase for each continuous variable.

### 3.8 Post‐mortem studies

**FIGURE 5:**
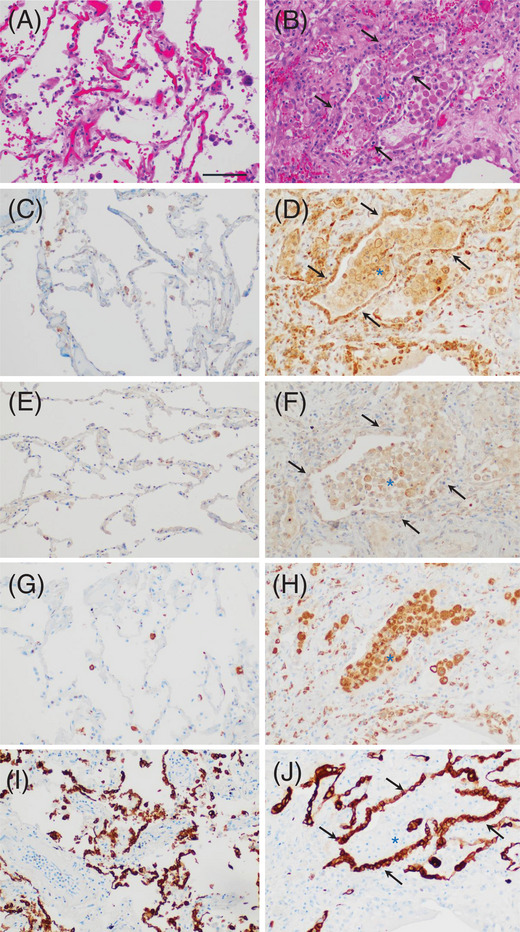
Upregulation of PAI‐1 and Neuroserpin in the lungs of patients with fatal SARS‐CoV‐2 infection, and controls. (A) Control case #1. H&E shows preserved alveolar structures. (B) COVID‐19 case #2. H&E shows loss of normal lung architecture and changes associated with the disease. The arrows indicate the alveolar epithelium. (C) Control case #1. PAI‐1 expression determined with anti‐PAI‐1 polyclonal antibody is minimally detected in the alveolar epithelium. (D) COVID‐19 case #2. Upregulation of PAI‐1 in epithelial cells (arrows) and macrophages (asterisk). (E) Control case #1. Anti‐Neuroserpin polyclonal antibody shows scattered macrophages, but is negative in other cell types. (F) COVID‐19 case #2. Expression of Neuroserpin in intra‐alveolar macrophages (asterisk) but not in epithelial cells (arrows). (G) Control case #1. Anti‐CD68 shows few intra‐alveolar macrophages. (H) COVID‐19 case #2. Anti‐CD68 reveals abundant intra‐alveolar macrophages (asterisk). (I) Control case #1. Anti‐cytokeratin antibody  stains alveolar epithelium. (J) COVID‐19 case #2. Anti‐cytokeratin highlights alveolar epithelium (arrrows). All images (x200). Bar represents 100 μm.

We studied four patients without COVID‐19 who died of conditions not associated with pulmonary disease, and 4 other patients who died from COVID‐19 with acute lung injury [19]. Table S1 describes specifics for each case, similar to prior studies [5, 19]. To investigate the fibrinolytic state in the lung, immunohistochemistry for PAI‐1 and Neuroserpin was studied using polyclonal antibodies. Figure 5A shows the H&E of Control case #1 with preserved lung architecture. Figure 5B reveals COVID‐19 case #2 with features associated with the disease, including a case with abundant intraalveolar macrophages [19]. Control case #1 exhibits a minimal expression of PAI‐1 in the epithelial cells of the alveolar wall, and focal expression in macrophages (Figure 5C). In contrast, COVID‐19 case #2 highlights an upregulation of PAI‐1 associated with the alveolar epithelium (arrows) and macrophages (Figure 5D), while staining in endothelial cells was variable/weakly positive (not shown). A semi‐quantitative score of “4” for PAI‐1 in the disease versus “2” for controls was statistically significant (Mann‐Whitney *U* test). For Neuroserpin staining, Control case #1 exhibits focal expression in macrophages (Figure 5E). In contrast, COVID‐19 case #2 shows strong staining in macrophages (Figure 5F) but not in other cell types. To confirm the histiocytic derivation for PAI‐1 and Neuroserpin staining, anti‐CD68 reveals few intra‐alveolar macrophages in Control case #1 (Figure 5G), while these were very abundant in COVID‐19 case #2 (Figure 5H). Neuroserpin expression was negative in epithelial cells and endothelial cells. A semi‐quantitative score of “4” for Neuroserpin in the disease versus “2” for controls was statistically significant (Mann‐Whitney *U* test). To confirm the epithelial staining for PAI‐1, Figure 5I shows cytokeratin immunostains (AE1/AE3) for the alveolar epithelium in control case#1, and Figure 5J marks the epithelium in COVID‐19 case #2. Endothelial cells were identified by morphology and with anti‐CD34 (not shown).

**FIGURE 6:**
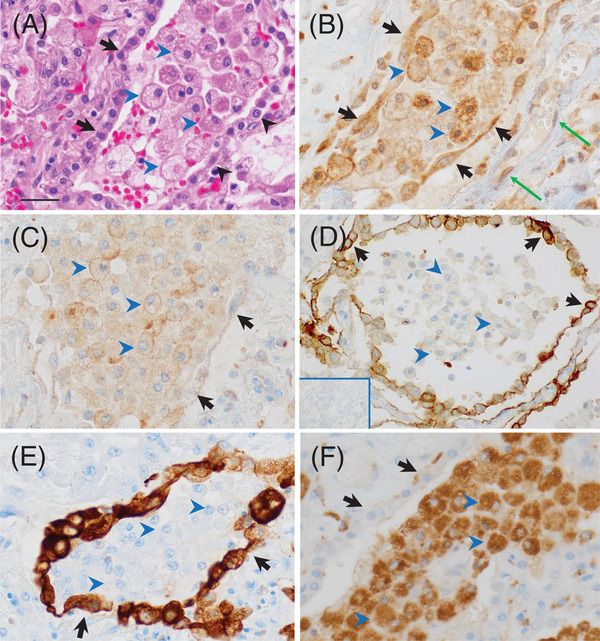
Cellular sources of PAI‐1 and Neuroserpin in the lung of SARS‐CoV‐2 infection. COVID‐19 case #2. (A) H&E shows the accumulation of reactive pneumocytes (short arrow) and intraalveolar macrophages (arrowhead) (x500). (B) Upregulation of PAI‐1 in epithelial cells (arrows) [29] and in macrophages (arrowheads) [30, 31, 32], with variable/weak staining in endothelial cells (long arrow) [33] (x500). (C) Neuroserpin expression in macrophages (arrowheads) is typically associated with the plasma membrane as reported [34], but not in epithelial cells (arrows) (x500). (D) As a comparison for staining specificity, anti‐tissue factor (TF) polyclonal antibody shows staining for TF in epithelial cells only (arrows), but not in macrophages (arrowheads)(x400), as reported [5]. The *inset* shows negative staining in the absence of a primary antibody. (E) Cytokeratin staining of epithelial cells with AE1/AE3 (arrows), but negative for macrophages (arrowheads) (x500). (F) CD68 confirms abundant intra‐alveolar macrophages (arrowheads) and is negative for epithelial cells (arrows) (x500). Bar represents 50 μm.

To illustrate the cellular sources for PAI‐1 and Neuroserpin in detail, high‐power views of the morphology and immunostain results for COVID‐19 case #2 are presented in Figure 6. Figure 6A shows the H&E of the alveolar epithelium and abundant intra‐alveolar macrophages. Figure 6B reveals marked staining for PAI‐1 in cells morphologically consistent with epithelial cells as reported [29], but also in macrophages [30, 31, 32], with variable/weak staining in endothelial cells [33]. Figure 6C shows punctate Neuroserpin staining typically concentrated in vesicles located in close proximity to the plasma membrane of macrophages, as reported [34]. In contrast, epithelial cells and endothelial cells were negative for Neuroserpin staining. To compare the specificity of our immunostains for PAI‐1 and Neuroserpin, with another polyclonal antibody, Figure 6D shows TF staining in epithelial cells only, but not in endothelial cells or macrophages, as reported [5]. In the absence of a primary antibody, staining was negative (Figure 6D, *inset*). To confirm cell derivations, Figure 6E shows the epithelium highlighted with AE1/AE3 antibody (for cytokeratin), and Figure 6F reveals the staining of macrophages with anti‐CD68.

## 4 DISCUSSION

SARS‐CoV‐2 infection results in hemostasis dysregulation and increased risk of thrombosis. However, it is unclear why most patients do not develop a consumption coagulopathy despite marked coagulation activation [1, 2, 3, 4]. Studying the repertoire of anticoagulants may be informative in this respect. Accordingly, our results show minimal changes in plasma concentrations of inhibitors of thrombin (AT, HCII, PN‐1), FXa (A2M and PZ), and kallikrein (Kallistatin) with no consumption of FXIII. Given the constant TAT levels [14], these results are indicative of low levels of ongoing systemic thrombin generation [9, 10]. Moreover, PC and the aPC inhibitor (PCI), and C1‐INH showed similar levels in patients and controls [21, 23, 35, 36]. The increase in plasma concentration of PZI (FXa inhibitor)[37], A1AT (inhibitor of neutrophil elastase) [21], and (total) PS (which binds to C4b‐Bp) was likely related to acute phase protein. There was a small elevation of PN‐2/AβPP (FXIa inhibitor) in moderate and severe disease, likely secondary to release by activated platelets [38]. Altogether, these results are in keeping with adequate regulation of the common, contact, and complement pathways at several steps by their corresponding inhibitors/regulators. These findings are typical for a non‐consumptive coagulopathy known as compensated DIC [39] and indicate that most of our patients are in this category. In these cases, a continuous or intermittent slow rate of initiation of intravascular coagulation occurs and control mechanisms (e.g., anticoagulants) may effectively prevent severe clinical manifestations, such as bleeding and hemorrhage [39], as opposed to overt DIC seen in sepsis, or in a subset of critically ill COVID‐19 patients [1, 2, 3, 4].

SARS‐CoV‐2 infection also presents with a hypofibrinolytic state despite high levels of D‐dimers; the mechanism(s) of this paradox is incompletely understood. Among four inhibitors of fibrinolysis evaluated (PAI‐1, PN‐1, A2AP, Neuroserpin), PAI‐1 was elevated in moderate and severe cases with a significant increase in t‐PAI/PAI‐1 complex formation, indicative of t‐PA neutralization. Additionally, PAI‐1 and t‐PA/PAI‐1 were associated with disease severity based on our univariate analysis, suggesting that PAI‐1 contributes to the pathogenesis of SARS‐CoV‐2 infection [40]. Of note, PAI‐1 is produced by adipose tissue and is elevated in obese patients [30, 41],  a morbidity associated with poor outcomes in the infection [1, 2, 3, 4]. Notably, our autopsy cases revealed overexpression of PAI‐1 in epithelial cells [29] and macrophages [30, 31, 32], with variable/dim staining in endothelial cells [33], indicating a high anti‐fibrinolytic activity in the lungs. This interpretation is supported by high PAI‐1 antigen levels detected in bronchoalveolar lavage samples collected from critically ill patients with SARS‐CoV‐2 infection and suppressed fibrinolysis by gene expression analysis [15, 36]. Of note, PAI‐1 is also present in large amounts in platelets, which are often detected as platelet‐rich thrombi in the pulmonary vessels of COVID‐19 infection [5], and in neutrophils [42]; both cells play a proinflammatory/procoagulant role in the disease [1–4, 43]. Furthermore, PAI‐1 is the principal fibrinolytic inhibitor in the pathogenesis of acute respiratory distress syndrome, representing an independent risk factor for poor prognosis and mortality in acute lung injury (ALI) [44, 45, 46], a condition that may develop COVID‐19 infection [1, 2, 3, 4]. Taken together, anti‐fibrinolytic activity through PAI‐1 expression takes place in the lungs through different cell types and conceivably contributes to circulating PAI‐1 levels leading to impaired systemic fibrinolysis.

Neuroserpin showed an increase in both moderate and severe cases of SARS‐CoV‐2 infection. Neuroserpin inhibits tPA, and to a lesser extent uPA and plasmin. Because Neuroserpin preferentially inhibits tPA, in contrast with PAI‐1 inhibits tPA and uPA, and preferentially localizes to neurons, it has been proposed that this serpin is the selective inhibitor of tPA in neurons. The mechanism(s) of Neuroserpin increase in the plasma of our patients is not clear, since the CNS typically produces Neuroserpin [47]. However, macrophages are potentially an important source, since these are among the myeloid cells known to produce this inhibitor [34]. Accordingly, our immunostain showed a typical pattern of expression of Neuroserpin [34] in intra‐alveolar macrophages where it accumulates in the lungs. Neuroserpin function is complex, with both vascular and cellular effects, and it is considered an endogenous neuroprotectant in the course of cerebral ischemia through plasmin‐dependent and independent mechanisms [47, 48, 49]. Interestingly, our results show that plasma Neuroserpin positively correlates with PAI‐1 and t‐PA/PAI‐1, and is associated with severity, suggesting participation in disease pathogenesis. However, a definitive role for Neuroserpin in venous thrombosis, stroke, and non‐ischemic neurologic abnormalities of SARS‐CoV‐2 infection remains to be determined [49, 50]. Finally, the results showed a modest but statistically significant increase in PN‐1 in severe cases only, which may negatively modulate fibrinolysis by inhibiting t‐PA [25].

Plasmin is the main enzyme that degrades the fibrin clot resulting in D‐dimer formation and is under inhibitory control of A2AP [27]. High levels of A2AP are associated with ischemic stroke in humans, and ischemic events in mice. The levels of A2AP remained constant in moderate and severe SARS‐CoV‐2, with an increase in PAP complex formation consistent with an activated fibrinolytic system, an interpretation also supported by an increase in t‐PA and D‐dimers in our cohort [5]. Elevated PAP also indicates plasmin inhibition and potential failure of fibrin dissolution, contributing to hypofibrinolysis. These findings are potentially important because clots are associated with trapped A2AP leading to fibrinolysis resistance, and circulating microclots are associated with long COVID‐19 [51]. Fibrinolysis is also modulated by TAFI, a carboxypeptidase that cleaves terminal lysine in fibrin, preventing t‐PA‐mediated plasminogen activation. We did not detect consumption of TAFI/TAFIa, in contrast to variable or increased levels reported in critically ill/ICU patients [14, 17, 20, 52], suggesting that TAFIa also functions as a negative modulator of fibrinolysis in advanced conditions.

Our results showed consumption of HRG, a cystatin‐like inhibitor involved in the regulation of coagulation/fibrinolysis by promoting the assembly of t‐PA with plasminogen leading to plasmin generation [28]. Low levels of HRG were associated with moderate and severe disease, suggesting a role in pathogenesis. HRG also negatively correlated with inhibitors of fibrinolysis (e.g. PAI‐1). In mice, HRG prevents septic lethality through negative regulation of immunothrombosis [28]. It is conceivable that reduced HRG contributes to hypofibrinolysis, and/or promotes inflammation in SARS‐CoV‐2 infection. Other mechanisms may explain fibrinolysis shutdown in the disease. SARS‐CoV‐2 spike protein S1 binds to fibrinogen and induces structurally abnormal blood clots with heightened proinflammatory activity and fibrin(ogen) resistant to fibrinolysis [53]. This lytic impairment may result in persistent large microclots [54]. It is plausible that local expression and systemic hypofibrinolysis by numerous inhibitors described above, in addition to abnormalities in the fibrin(ogen) molecule, contribute to a hypofibrinolytic state typical of COVID‐19 patients. This interpretation is supported by numerous TEG results showing impaired fibrinolysis in SARS‐CoV‐2 and its association with thrombosis [11, 13, 14, 40].

Altogether, a picture emerges where two compartments contribute to hemostasis dysregulation in the disease. The lung (“first compartment”) is the epicenter of immunothrombosis by numerous mechanisms, resulting in a heightened coagulation‐inflammation cycle. Accordingly, intermittent/localized coagulation factor activation induced by pulmonary TF expression, and NETs formation (among others) overcomes inhibition by local anticoagulants whose expression and functions are downregulated or impaired due to endothelial cell damage (e.g., thrombomodulin). This results in abundant pulmonary fibrin deposition and platelet thrombi [1, 2, 3, 4, 5, 6, 7]. Increased D‐dimers, produced by compensatory fibrinolysis, reaches the systemic circulation (“second compartment”) where activated coagulation factors are neutralized by their respective inhibitors. This explains the lack of consumption coagulopathy together with elevated D‐dimers. Additionally, high levels of fibrinolytic inhibitors (e.g., PAI‐1) released by platelets and endothelial cells in the lung and elsewhere, together with Neuroserpin, PN‐1, TAFI, A2AP, and reduced HRG, redundantly inhibit t‐PA and/or plasmin generation and/or function. This explains a systemic hypofibrinolytic state, despite elevated D‐dimers. This process may be exacerbated by Virchow's triad (hypercoagulability, endothelium activation, venous stasis), causing the endothelial release of von Willebrand factor multimers that may facilitate platelet‐vessel wall interactions and platelet‐rich thrombosis in the microvasculature, providing the substrate for venous (and occasionally arterial) thrombosis. Acquired or inherited thrombophilia may also contribute to thrombotic events in some cases [55, 56, 57]. In patients who are critically ill, marked activation of the coagulation cascade promotes a decompensated response resembling overt DIC seen in sepsis [8, 9, 55].

One important limitation of this study is that results and conclusions may not be generalizable to all critically ill/ICU patients in whom a decompensated state (overt DIC) may develop. Another limitation is that most patients were on prophylactic heparin, which may have prevented coagulation dysregulation to a certain extent. Although our results by ELISA are in excellent agreement with proteome analysis of SARS‐CoV‐2 plasma/serum for several inhibitors (e.g., Kallistatin, PZI, A2M, HCII, PS, A1AT, and HRG) [35, 58, 59], our analysis was limited to the concentration of inhibitors in the plasma; therefore, type II deficiencies (antigen levels are normal, but the activity levels are low) cannot be excluded. Despite these limitations, the marked increase in anti‐fibrinolytic molecules in the lung and in the circulation suggests that going forward, targeting fibrinolysis may be a useful therapeutic strategy when combined with other modalities [60].

## AUTHOR CONTRIBUTIONS

Authors participated in study design (Kevin H. Toomer, Gloria F. Gerber, Yifan Zhang, Jody E. Hooper, Ivo M. B. Francischetti), data analysis (Kevin H. Toomer, Gloria F. Gerber, Yifan Zhang, Laetitia Daou, Jody E. Hooper, Ivo M. B. Francischetti), sample collection and experimentation (Kevin H. Toomer, Gloria F. Gerber, Yifan Zhang, Michael Tushek, Jody E. Hooper, Ivo M. B. Francischetti), and writing (Kevin H. Toomer, Gloria F. Gerber, Yifan Zhang, Jody E. Hooper, Ivo M. B. Francischetti).

## CONFLICT OF INTEREST STATEMENT

The authors declare no conflict of interest.

## FUNDING INFORMATION

This study was supported by the John Hopkins University School of Medicine grant 80053630 (Ivo M. B. Francischetti). Grant Number UL1TR003098 (Yifan Zhang). American Society of Hematology, Research Training Award for Fellows: 140989 (Gloria Gerber).

## AVAILABILITY STATEMENT

The data that support the findings of this study are available upon request from the corresponding author. The data are not publicly available due to privacy or ethical restrictions.

## ETHICS STATEMENT

All procedures were in accordance with the ethical standards of the respective local research committee and with the 1964 Helsinki declaration and its later amendments or comparable ethical standards.

## Supporting information




**Table S1**. Demographic and clinical information for autopsy cases.
